# *MBOVJF4278_00820* encodes a novel cytoadhesin of *Mycoplasma bovis* binding to heparin

**DOI:** 10.1128/iai.00606-24

**Published:** 2025-04-23

**Authors:** Qi Wu, Zhixin Ma, Qiao Pan, Tong Liu, Jiuqing Xin, Qingyuan Xu

**Affiliations:** 1 State Key Laboratory for Animal Disease Control and Prevention, Harbin Veterinary Research Institute, Chinese Academy of Agricultural Sciences, Harbin, Heilongjiang, China; 2 Institute of Western Agriculture, Chinese Academy of Agricultural Sciences, Xinjiang, China; Rutgers-New Jersey Medical School, Newark, New Jersey, USA

**Keywords:** *Mycoplasma bovis*, adhesin, heparin, immunogenicity

## Abstract

*Mycoplasma bovis*, a minimal bacterium but a notorious cattle pathogen, leads to serious economic losses. This pathogen binding to host cells is emerging as a complex process involving a broad range of surface-exposed structures. For mycoplasma, adhering to host tissue is the first and crucial step of infection. It is well known that the molecules contributing to microbial cytoadhesion are important virulence factors. Here, we cloned, expressed, and purified the recombinant protein, which is encoded by *MBOVJF4278_00820*, and induced polyclonal antibodies for it in mice. The cytoadhesive properties of this surface-exposed protein were demonstrated on embryonic bovine lung (EBL) and Madin-Darby bovine kidney (MDBK) cells. Furthermore, heparin as the binding target was confirmed, and the characteristics of the interaction between this protein and heparin have also been analyzed. Our data indicate that the surface-associated *MBOVJF4278_00820*-encoded protein is a novel adhesion-related factor of *Mycoplasma bovis*.

**IMPORTANCE:**

Adhesins are crucial in facilitating *Mycoplasma bovis* infection. In this study, we identified a specific *Mycoplasma bovis* adhesin that interacts with heparin on the surface of host cells. Given that heparin is ubiquitously distributed across a wide range of tissue cells, the identification of the heparin-binding adhesin is significant for elucidating how *Mycoplasma bovis* targets diverse host cells and triggers a spectrum of clinical manifestations.

## INTRODUCTION

*Mycoplasma bovis* (*M. bovis*) is an infamous animal pathogenic microorganism that can induce bronchopneumonia, mastitis, arthritis, keratoconjunctivitis, and genital diseases in infected cattle (1, 2). This pathogenic microorganism can be co-infected with various pathogens such as *Pasteurella multocida* (3), *Haemophilus somnus* (4), bovine respiratory syncytial virus (5), bovine herpesvirus 1 (6), bovine viral diarrhea virus (4), *Histophilus somni* (7), and parainfluenza virus type 3 (5), leading to an aggravation of the disease in infected animals. Furthermore, *M. bovis* is also one of the main pathogens of the bovine respiratory disease complex (8, 9). Considering the harmfulness of *M. bovis*, it is receiving increasing attention. However, *M. bovis* virulence mechanisms are still poorly understood, although tremendous efforts have been expended.

Adhesion to host tissues and cells is a prerequisite for bacterial colonization and virulence, and for mycoplasma, it is also widely accepted that the first step in infection is to adhere to host cells. In reality, this process may be complex, and for a single microbe, it can involve redundancy or the possession of multiple adhesins or mechanisms of attachment (10). The importance of the diversity of receptor targeting mechanisms to the success of adherence and subsequent pathogenesis is evident, because many bacteria may use phase variation, displaying on-off expression of different surface structures for immune evasion. Through the continuous efforts of researchers, various adhesins of *M. bovis* have been identified. For example, the adhesins, with definite host binding targets, including NOX (11), MbfN (12), FBA (13, 14), TrmFO (15), α-Enolase (16), MilA (17), and P27 (18), LppA (19), LppB (20), as well as VpmaX (21), P26 (22), VSPs (23, 24), Mbov-0503 (25), and the 24kDa-protein (26), which are not yet clear about host target proteins. Unfortunately, for *M. bovis*, although more than 10 types of adhesins have been identified, none of them can completely block the adhesion of the bacteria to the host cell. This means that the adhesion between *M. bovis* and host cells is the result of the combined action of multiple adhesins.

The binding targets of bacterial adhesins are the main components on the host cell surface, including collagen, elastin, fibronectin, plasminogen, laminin, heparin, platelet-derived growth factors, and so on (27, 28). Currently, four types of *M. bovis* adhesin binding components have been identified, comprising fibronectin, plasminogen, heparin, and amyloid precursor-like protein-2 (29). Among these adhesive binding targets, heparin is a special one. As a widespread sulfated glycosaminoglycan, heparin sulfate is widely present in all types of tissues and cells at the extracellular and cellular levels. The heparin sulfate chains are synthesized in the Golgi apparatus, and the multiple binding activities of heparin sulfate are closely linked to its extended structural variability. The heparin sulfate chain has great diversity, and this provides heparin sulfate chains with different docking sites for various ligands of polysaccharides. It is reported that various pathogens use heparin sulfate proteoglycan as the target for infection, and the binding of pathogenic ligands to heparin is closely related to pathogen internalization and pathogenesis (10).

To identify the adhesins of *M. bovis*, we conducted an interaction between whole-cell lysates of *M. bovis* and embryonic bovine lung (EBL) and Madin-Darby bovine kidney (MDBK) cells, which led to the selection of several candidate adhesin proteins. In this study, a novel adhesin of *M. bovis* was identified by a variety of biological and immunological methods. The cytoadhesion protein encoded by the *MBOVJF4278_00820* gene was found to facilitate the binding of *M. bovis* to host cells and further demonstrated that this protein achieves adhesion function by binding to heparin. All of these data make it a potential virulence factor to be further studied.

## RESULTS

### Expression, immunogenicity, and subcellular localization identification of *MBOVJF4278_00820***-encoded protein**

**Fig 1:**
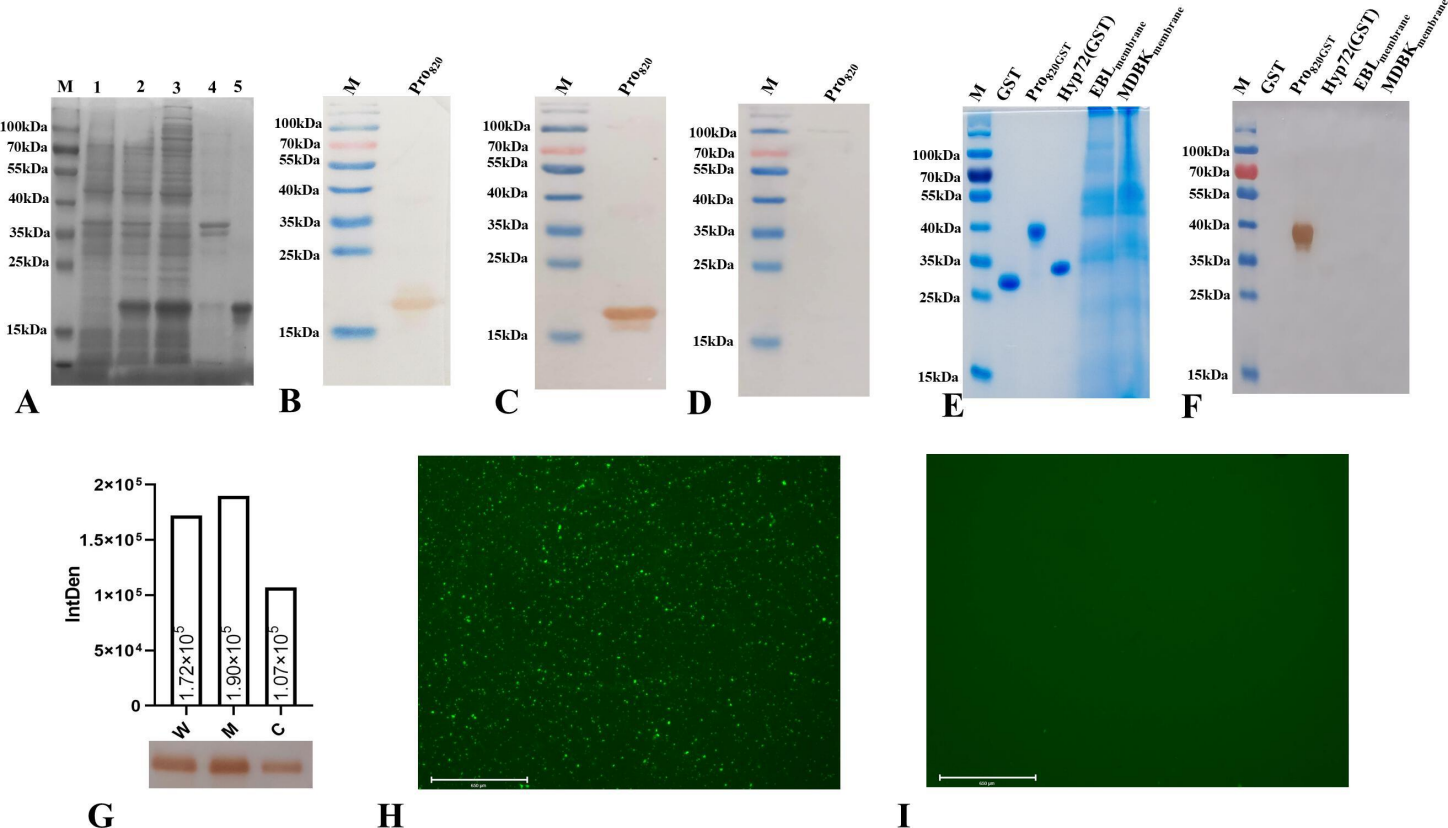
(A) SDS-PAGE analysis of the induced expression protein revealed that compared to the pET28a (+) empty vector (line 1), the *MBOVJF4278_00820* gene recombinant bacteria showed significant bands (line 2) between 15 kDa and 25 kDa after induction. The expressed protein was mainly soluble (cell lysis supernatant) (line 3), and only a small amount of protein was precipitated in cell lysis precipitation (line 4). The expressed protein could be effectively purified (line 5). The lane M is a protein marker; (**B**) the purified protein was transferred to a nitrocellulose film for anti-HIS labeled antibody detection, and clear bands were observed between 15 kDa and 25 kDa; (**C**) the Pro820 protein reacts with *M. bovis*-positive serum; (**D**) the Pro820 protein was not recognized by *M. bovis*-negative serum; (**E**) the input samples for mouse anti-Pro820 protein serum specificity identification; (**F**) the mouse anti-Pro820 protein serum is characterized for recognizing Pro820 protein and does not react with GST tag, GST tag-unrelated proteins, or membrane components of MDBK cells and EBL cell membranes; (**G**) western blotting analysis showed that the bands of *MBOVJF4278_00820*-encoded protein were identified in whole cell proteins, cell membrane proteins, and cytoplasmic proteins. The intensity of the western blotting reaction is semi-quantified by grayscale values; indirect immunofluorescence analysis with anti-Pro820 polyclonal antibodies for subcellular localization; (**H**) the positive serum group showed significant green fluorescence (Bar = 650 µm); (**I**) there was no specific fluorescence observed in the negative serum group (Bar = 650 µm).

Using the genome of *M. bovis* strain TJ as a template, the *MBOVJF4278_00820* gene was amplified with specific primers and cloned into a pET28a (+) vector to construct a prokaryotic expression plasmid. After correct sequencing, it was transformed into BL21 (DE3) competent cells for induced expression, and the recombinant protein of Pro820 was obtained. SDS-PAGE was used to identify the protein expression, and the results showed this protein was successfully expressed. The expressed protein mainly existed in soluble form, and the expressed protein could be effectively purified (Fig. 1A). The purified protein was identified using anti-HIS labeled monoclonal antibodies, and then a clear band was observed between 15 kDa and 25 kDa (Fig. 1B).

Western blotting was used to identify the immunogenicity of Pro820. The purified recombinant protein was transferred to the NC film and incubated with *M. bovis* positive serum and negative serum, respectively. After the color reaction, it was found that *M. bovis* positive serum can react with Pro820 (Fig. 1C), while negative serum cannot recognize this protein (Fig. 1D). This result indicates that cattle infected with *M. bovis* can produce antibodies against Pro820, which means that this protein is an immune component of *M. bovis*.

After immunizing BALB/c mice with the Pro820 protein, the resulting anti-Pro820 hyperimmune serum with a titer of 1:405,000 was obtained. The results of western blotting indicate that this serum specifically recognizes the untagged Pro820 without reacting with the GST tag, unrelated proteins fused with the GST tag, and the membrane component of host cells (Fig. 1E and F).

To identify the subcellular localization of the *MBOVJF4278_00820*-encoded protein in *M. bovis*, the whole cell proteins, membrane proteins, and cytoplasmic proteins of *M. bovis* were analyzed using mouse anti-Pro820 serum. Western blotting results show that the anti-Pro820 protein detected a single band of consistent size in all three samples, indicating that the protein encoded by the *MBOVJF4278_00820* gene is present in the whole cell, membrane, and cytoplasm of *M. bovis* (Fig. 1G). Due to the inability of western blotting to completely rule out the possibility of membrane protein contamination by cytoplasmic proteins, we further confirmed the surface localization of the *MBOVJF4278_00820*-encoded protein in *M. bovis* using indirect immunofluorescence assay (IFA). After the reaction between *M. bovis* and anti-Pro820 serum, a clear green fluorescence signal can be observed under a fluorescence microscope (Fig. 1H), while the fluorescence signal is not observed in pre-immune mouse serum (Fig. 1I). The above experiments all indicate that the *MBOVJF4278_00820*-encoded protein is located on the surface of *M. bovis*.

### Pro820 adheres to EBL and MDBK cells

**Fig 2:**
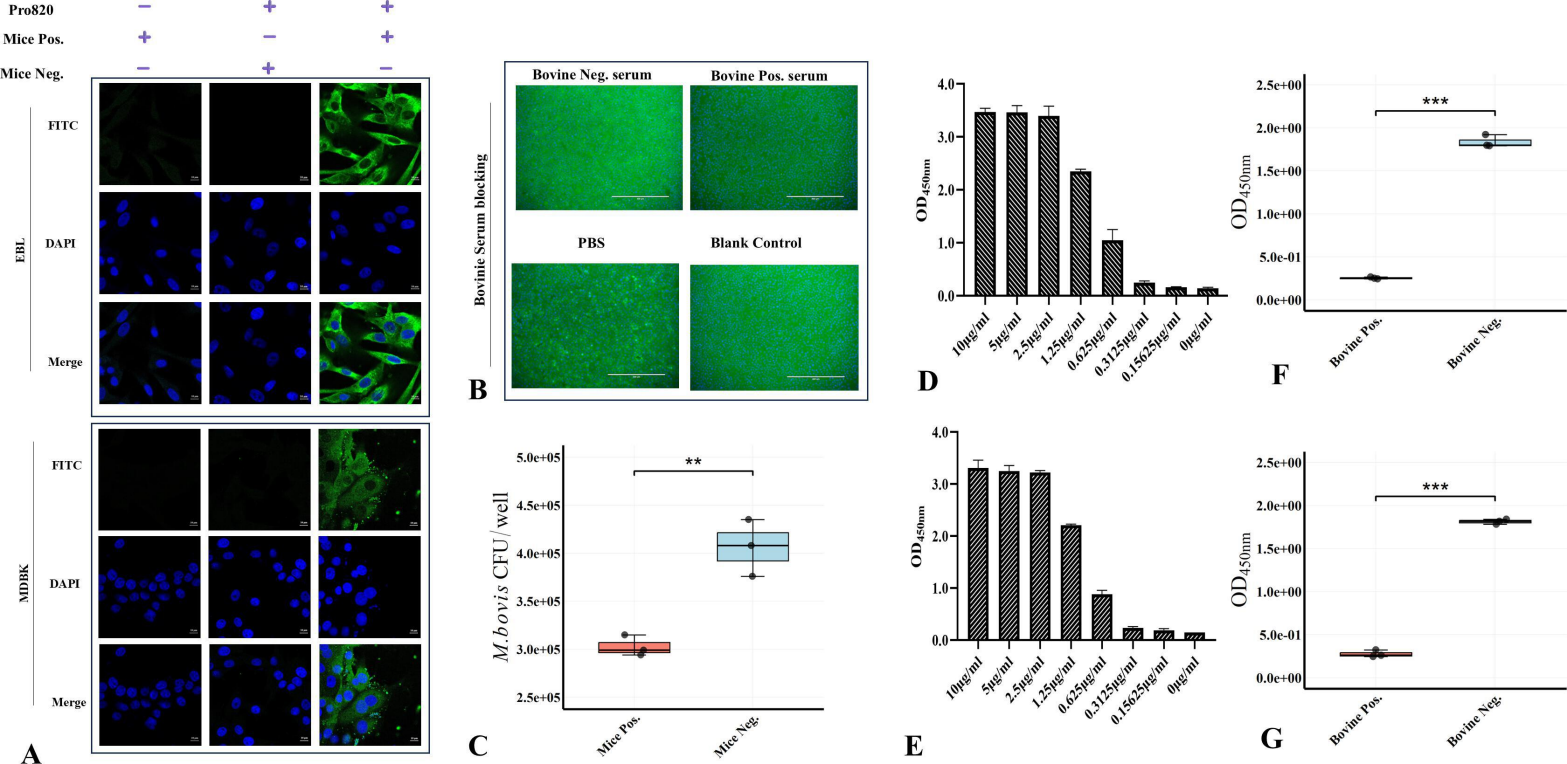
(A) Confocal microscopy analysis of Pro820 adhesion to host cells. The EBL cells and MDBK cells were incubated with Pro820 and tested for anti-Pro820 antibodies, conspicuous green fluorescence was observed. While no specific green fluorescence was examined in PBS control and negative serum groups (Bar = 10 µm); (B) the *M. bovis*-positive blocked the adhesion of the Pro820 protein to the host cells (Bar = 400 µm); (C) the anti-Pro820 polyclone antibodies partially reduced the adhesion of *M. bovis* to host cells (** present *P* < 0.01); (D) the adhesion of the Pro820 to EBL cell membrane components was confirmed by enzyme-linked immunosorbent assay (ELISA), the reaction showed dose-dependent manner and saturation adhesion at a concentration of 2.5 µg/mL; (E) the adhesion of the Pro820 to MDBK cell membrane extracts was dose-dependent, and the binding reached saturation at a concentration of 2.5 µg/mL; analyze the effect of *M. bovis*-positive serum on the Pro820 binding to host cell membrane components by ELISA; (F) the binding of Pro820 to the membrane components of EBL was significantly inhibited by 50-fold dilution positive serum compared to negative serum control (*** present *P* < 0.001); (G) the Pro820 binding to MDBK membrane components was significantly reduced for inhibiting with 50-fold positive serum (*** present *P* < 0.001).

Confocal laser scanning microscopy was employed to visualize the adhesion of Pro820 to EBL cells and MDBK cells. As shown in Fig. 2, after co-incubation of Pro820 with EBL cells and MDBK cells, significant green fluorescence was observed in EBL cells and MDBK cells using anti-Pro820 serum detection (Fig. 2A). However, no specific fluorescence was found in the pre-immune serum group and the non-recombinant protein group. This result indicates that Pro820 can adhere to both EBL and MDBK cells.

### Positive serum of *M. bovis* reduces the adherence of Pro820 protein to host cells

To further validate the binding of the Pro820 protein to host cells, we utilized MDBK cells as a representative model. Employing naturally infected *M. bovis*-positive serum, we successfully blocked the adhesion of Pro820 protein to the host cells. Our results demonstrated that the positive serum, diluted at a ratio of 1:10, potently hindered the interaction of Pro820 with MDBK cells. This outcome provides additional evidence that the Pro820 protein is capable of engaging with surface components on MDBK cells (Fig. 2B).

### Anti-Pro820 serum inhibits *M. bovis* adhesion to cells

To evaluate the role of the *MBOVJF4278_00820*-encoded protein in *M. bovis* infection, the inhibitory effect of anti-Pro820 serum on the adhesion to host cells was analyzed using MDBK cells as a model. Incubate *M. bovis* with anti-Pro820 serum or pre-immune serum, then infect MDBK cells, harvest infected cells, and inoculate them with a solid culture medium to count colonies. Compared with the pre-immune serum treatment group, the positive serum treatment group significantly reduced the number of bacterial colonies (Fig. 2C). This result indicates that anti-Pro820 serum can partially reduce the adhesion of *M. bovis*.

### Pro820 interacts with cell surface components

To verify the interaction between the *MBOVJF4278_00820*-encoded protein and surface components of EBL and MDBK cells, the cell membrane components of EBL and MDBK cells were analyzed for adhesion to the Pro820 using the enzyme-linked immunosorbent assay (ELISA) method. After coating 10 µg/mL membrane proteins onto an ELISA plate, purified Pro820 at different concentrations reacted with it. For the membrane components of these two types of cells, the Pro820 reached the plateau at a concentration of 2.5 µg/mL, while after 0.3125 µg/mL, the OD value approached the background level (Fig. 2D and E). This result indicates that the Pro820 can react with the membrane components of EBL cells and MDBK cells, and this adhesion exhibits a dose-dependent feature.

To ascertain the impact of *M. bovis*-positive serum on the interaction between the *MBOVJF4278_00820*-encoded protein and components of the host cell membrane, an adhesion inhibition assay was conducted employing ELISA methodology. Compared with negative serum, a 50-fold dilution of *M. bovis* positive serum can significantly inhibit the adhesion of Pro820 to the membrane components of EBL cells and MDBK cells (Fig. 2F and G).

### Pro820 interacts with heparin

**Fig 3:**
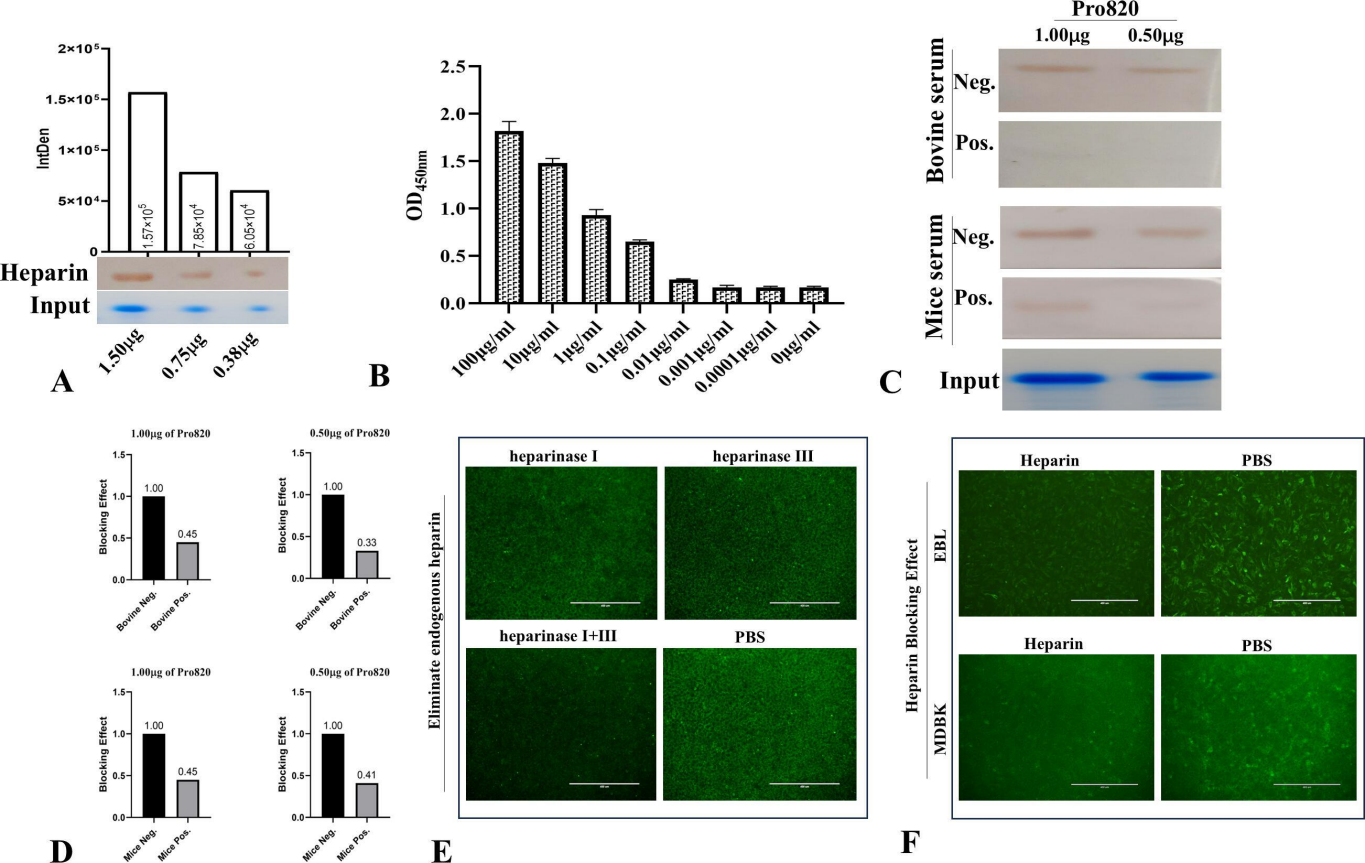
(A) The western blotting experiment confirmed that different concentrations of Pro820 can directly interact with heparin. The intensity of the western blotting reaction is semi-quantified by grayscale values; (**B**) analyze the interaction between Pro820 and heparin using ELISA. Using Pro820 as the coating antigen and incubating with different concentrations of heparin, it was found that the heparin and the Pro820 can interact with each other, and the reaction occurs in a dose-dependent manner; (**C**) western blotting validation of the effects of natural infection serum of *M. bovis* and mouse anti-Pro820 serum on the reaction between various concentrations of the Pro820 and heparin. Compared to the negative sera, both bovine-positive serum and mice anti-Pro820 serum can block the reaction between the Pro820 and heparin; (**D**) using Image J to analyze the reaction intensity of western blotting reactions where the interaction between the Pro820 and heparin is blocked by *M. bovis* naturally infected serum and mouse anti-Pro820 protein serum. The blocking effect observed with the negative serum will serve as the baseline reference for illustrating the comparative blocking efficacy of the positive serum under various experimental conditions; (**E**) after heparinase treatment to remove heparin from MDBK cell-derived sources, the ability of the Pro820 to adhere to host cells was significantly reduced (Bar = 400 µm); (**F**) following the incubation of the Pro820 protein with heparin, a noticeable reduction in its adherence to EBL and MDBK cells was detected (Bar = 400 µm).

Heparan is a widespread form of sulfated glycosaminoglycan, which is present in all types of tissues and cells at extracellular and cellular levels, and many pathogens can react with heparin on the surface of host cells. To identify whether the *MBOVJF4278_00820*-encoded protein can bind to heparin, the interaction between Pro820 and heparin was analyzed using western blotting. The results indicated that heparin can bind to the Pro820 protein that is immobilized on the NC film. After diaminobenzidine (DAB) substrate coloration, a distinct band appeared at the expected location, and the intensity of the band was found to be directly proportional to the amount of Pro820 protein (Fig. 3A). This result indicates that the *MBOVJF4278_00820*-encoded protein binds to heparin on the cell surface, thereby assisting in pathogen adhesion.

To further validate the interaction between the *MBOVJF4278_00820*-encoded protein and heparin, the Pro820 was coated on an ELISA plate and reacted with different concentrations of biotinylated heparin. ELISA experiments also confirmed that the Pro820 protein can bind to heparin, and this binding exhibits a dose-dependent relationship. As the concentration of heparin decreased, the OD values in the corresponding wells on the ELISA plate decreased accordingly (Fig. 3B).

### Adhesion inhibition assay for Pro820 and heparin

To analyze the blocking effect of *M. bovis* positive serum on the *MBOVJF4278_00820*-encoded protein adhesion, we used positive serum from naturally infected cattle with *M. bovis* TJ strain to block the reaction between Pro820 and heparin. Two concentrations of Pro820 were transferred to the NC film. Before reacting with heparin, *M. bovis* infected cattle-positive serum was incubated with the Pro820, and negative serum was set as a control. The results showed that the serum of naturally infected cattle can significantly block the binding of Pro820 to heparin (Fig. 3C). Western blotting grayscale analysis results indicated that, compared to the negative serum, after blockade with the positive serum, the protein adhesion capacity of the 1 µg group and the 0.5 µg group decreased by 55% and 67%, respectively (Fig. 3D).

It was also analyzed using the same method as *M. bovis* positive serum, the effect of mouse anti-Pro820 protein antibodies on the binding of Pro820 to heparin. The results showed that anti-Pro820 antibodies could also inhibit the binding of this protein to heparin; the inhibitory ability was almost equal to that of positive serum of *M. bovis* (Fig. 3C), and the protein adhesion capacity of the 1 µg protein and the 0.5 µg group decreased by 55% and 59%, respectively (Fig. 3D).

### Removing endogenous heparin reduces the adherence of Pro820 protein to host cells

Heparinase I and III can decompose sodium heparin or heparin sulfate into unsaturated uronic acid, respectively. To further validate how *MBOVJF4278_00820*-encoded protein interacts with heparin of the cell surface. We used MDBK cells as a model and removed the endogenous heparin from the cell surface using heparinase I and heparinase III. Treatment with heparinase I or III alone shows a minor effect on the adhesion of Pro820 to MDBK cells. But if both of the two heparinases are incubated with MDBK cells simultaneously, the binding of Pro820 to MDBK cells is reduced significantly (Fig. 3E). This result confirmed that the Pro820 indeed binds to heparin on the surface of host cells, and the Pro820 may have the ability to react with different styles of heparin located on the surface of host cells.

### Heparin blocked the adhesion of Pro820 to host cells

If heparin plays a role in the adhesion of *MBOVJF4278_00820*-encoded proteins to host cells, pre-treatment with heparin may affect the adhesion effect of this protein on host cells. To verify this hypothesis, we conducted an adhesion inhibition experiment by pre-treating Pro820 protein with heparin. The results showed that although complete blockade could not be achieved, pre-treatment with heparin did indeed affect the adhesion of Pro820 to host cells, and this blocking effect was observed in both EBL and MDBK cells (Fig. 3F). This further indicates that the *MBOVJF4278_00820*-encoded protein can achieve adhesion to different host cells through heparin.

### Identification of binding regions to heparin and epitope for positive serum

**Fig 4:**
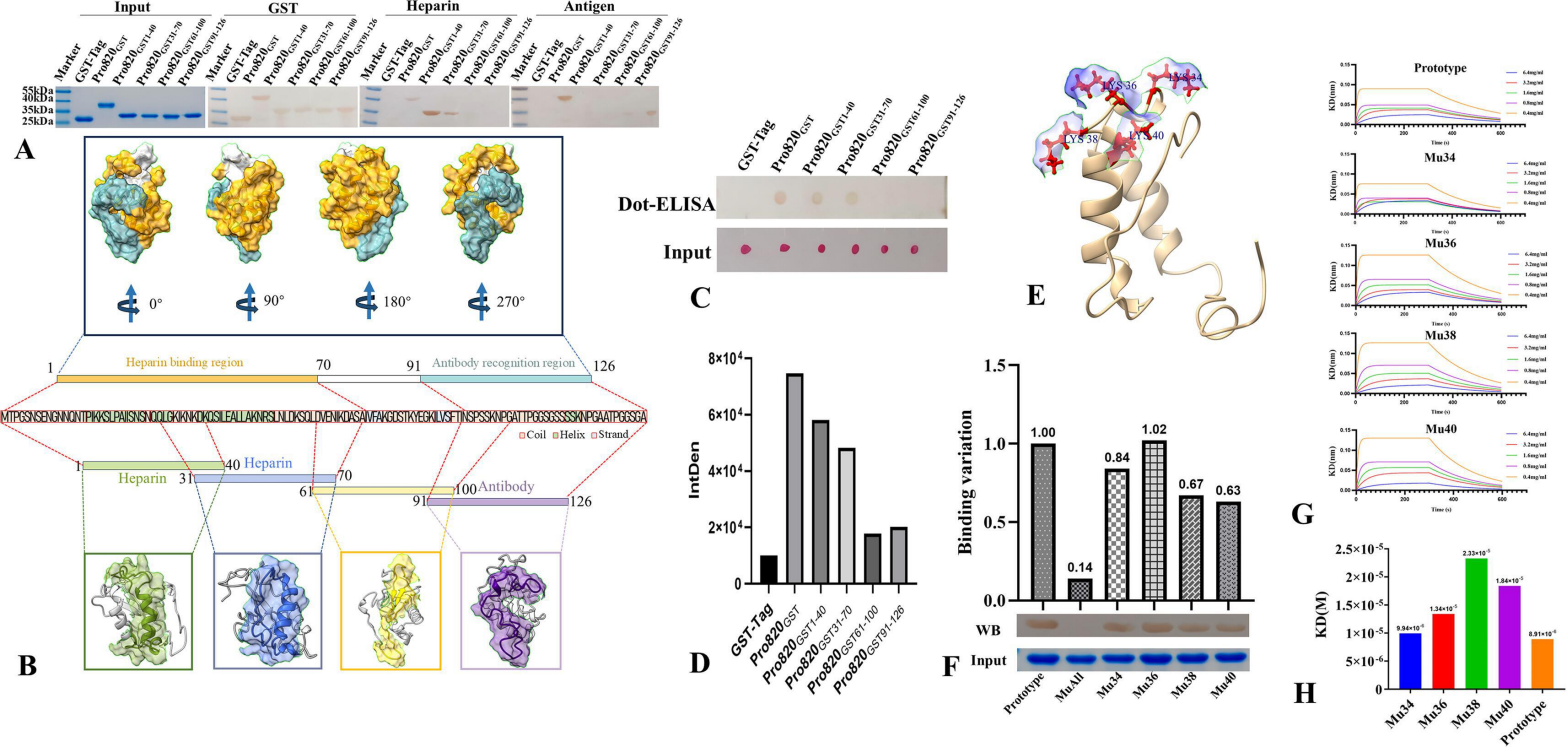
(A) Western Blotting verifies the region where the Pro820 reacts with heparin and *M. bovis* naturally infected serum. Lines 1 to 6 represent equal amounts of each protein sample used for analysis. Lines 7 to 12 were detected by anti-GST antibody. Lines 13 to 18 reacted with heparin. Lines 19 to 24 reacted with the positive serum of *M. bovis*; (**B**) analyze the heparin-binding characteristics, antibody-binding regions, and structural characteristics of the *MBOVJF4278_00820*-encoded protein through its tertiary structure. The heparin reaction is mainly located between the 1 and 70 amino acids, while the antibody binding reaction region is located between the 91 and 126 amino acids; (**C**) dot-ELISA was used to analyze the reactivity of different fragments of the Pro820 with heparin under non-denaturing conditions, and the protein samples used for analysis were simultaneously stained with Ponceau S; (**D**) dot-ELISA result for the heparin reactivity of the Pro820 was analyzed by grayscale analysis with Image J software; (**E**) the lysines at positions 34, 36, 38, and 40, located within the reactive area, are on the protein surface, with blue color indicating that the surface area carries a positive charge; (**F**) take the Pro820_1-70GST_ as prototype, the western blotting analysis was conducted to examine the effect of mutations at lysine residues 34, 36, 38, and 40 on heparin binding. The reactivity of different samples was semi-quantitatively analyzed using the unmutated protein as a baseline; (**G**) bio-layer interferometry (BLI) analysis was conducted to assess the affinity of the unmutated protein and various mutants, where lysine was replaced with alanine, for heparin; (**H**) the impact of lysine mutations at positions 34, 36, 38, and 40 on the affinity for heparin.

To further analyze the heparin-binding characteristics and antigenicity of the *MBOVJF4278_00820*-encoded protein, the protein was submitted to the I-TASSER online server for structural prediction. At the same time, this protein was expressed in whole or truncated to identify the regions that react with heparin. Western blotting analysis revealed that the first segment (comprising amino acid residues 1–40) and the second segment (spanning amino acid residues 31–70) of the protein encoded by the *MBOVJF4278_00820* gene exhibited a reaction with heparin. In contrast, the remaining portions failed to interact with heparin. These findings suggest that the amino acid sequence from 1 to 70 constitutes the heparin-reactive domain (Fig. 4A). To avoid the impact of structural loss on the western blotting results, dot-ELISA was used to verify the binding ability of the above protein fragments to heparin. Under non-denature conditions, the first two fragments also reacted with heparin, while the other two fragments did not. This result further proves that the first 70 amino acids of the *MBOVJF4278_00820*-encoded protein are crucial regions for the reaction with heparin (Fig. 4C and D).

The segmented expression of the *MBOVJF4278_00820*-encoded protein was transferred to the NC film and then reacted with *M. bovis* naturally infected serum. The results showed that the fourth segment (91 to 126 amino acids) reacted with the positive serum (Fig. 4A).

The heparin reaction region and antibody binding region were analyzed on the tertiary structure of the protein, and the results showed that the two regions did not overlap, but they were closely connected. From a structural perspective, heparin looks more inclined to bind to the α-Helix region of the *MBOVJF4278_00820*-encoded protein, while antibodies are more likely to react with random coils (Fig. 4B).

### Identification of key amino acids involved in heparin interaction

After truncation, the 1–40 amino acid region and the 31–70 amino acid region of the *MBOVJF4278_00820*-encoded protein can react with heparin. This suggests that the overlapping part, the 31–40 amino acid region, may contain key amino acids that interact with heparin. Heparin is a negatively charged polymer, and positively charged amino acid residues in heparin-binding proteins are crucial for binding to heparin (30). Indeed, within the 31–40 amino acid region, there exist four positively charged lysines situated on the protein surface and located at positions 34, 36, 38, and 40 (Fig. 4E). Accordingly, we expressed the first 70 amino acid region of the *MBOVJF4278_00820*-encoded protein and constructed a mutant in which all four lysines were replaced by alanines. Western blotting analysis revealed that the mutated protein lost its ability to react with heparin (Fig. 4F). In order to analyze the role of these four amino acids in the heparin-binding process, we used Pro820_1-70GST_, which contained the first 70 amino acids of the *MBOVJF4278_00820*-encoded protein, as a template to mutate them respectively. However, any single lysine mutation did not result in the complete loss of the heparin-binding ability of Pro820_1-70GST_ (Fig. 4F). To further analyze the impact of mutations in these lysines on heparin binding, we used bio-layer interferometry (BLI) to explore the binding capacity of the unmutated protein and each single lysine mutant to heparin (Fig. 4G). The dissociation constant (KD) of Pro820_1-70GST_ with heparin was 8.91 × 10^−6^ M (Fig. 4H). After individually mutating lysines at positions 34, 36, 38, and 40 of Pro820_1-70GST_ to alanine, the affinities were 9.94 × 10^−6^, 1.34 × 10^−5^, 2.33 × 10^−5^, 1.84 × 10^−5^, and 8.91 × 10^−6^ M, respectively (Fig. 4H). Except for the amino acid at position 34, mutations in the other amino acid residues resulted in a significantly increased KD value of Pro820_1-70GST_ with heparin. These results indicate that lysines at positions 34, 36, 38, and 40 of the *MBOVJF4278_00820*-encoded protein are key amino acid residues involved in the reaction with heparin, with lysines at positions 36, 38, and 40 playing a more important role in the binding to heparin.

### *MBOVJF4278_00820*-encoded protein conservative analysis

**Fig 5:**
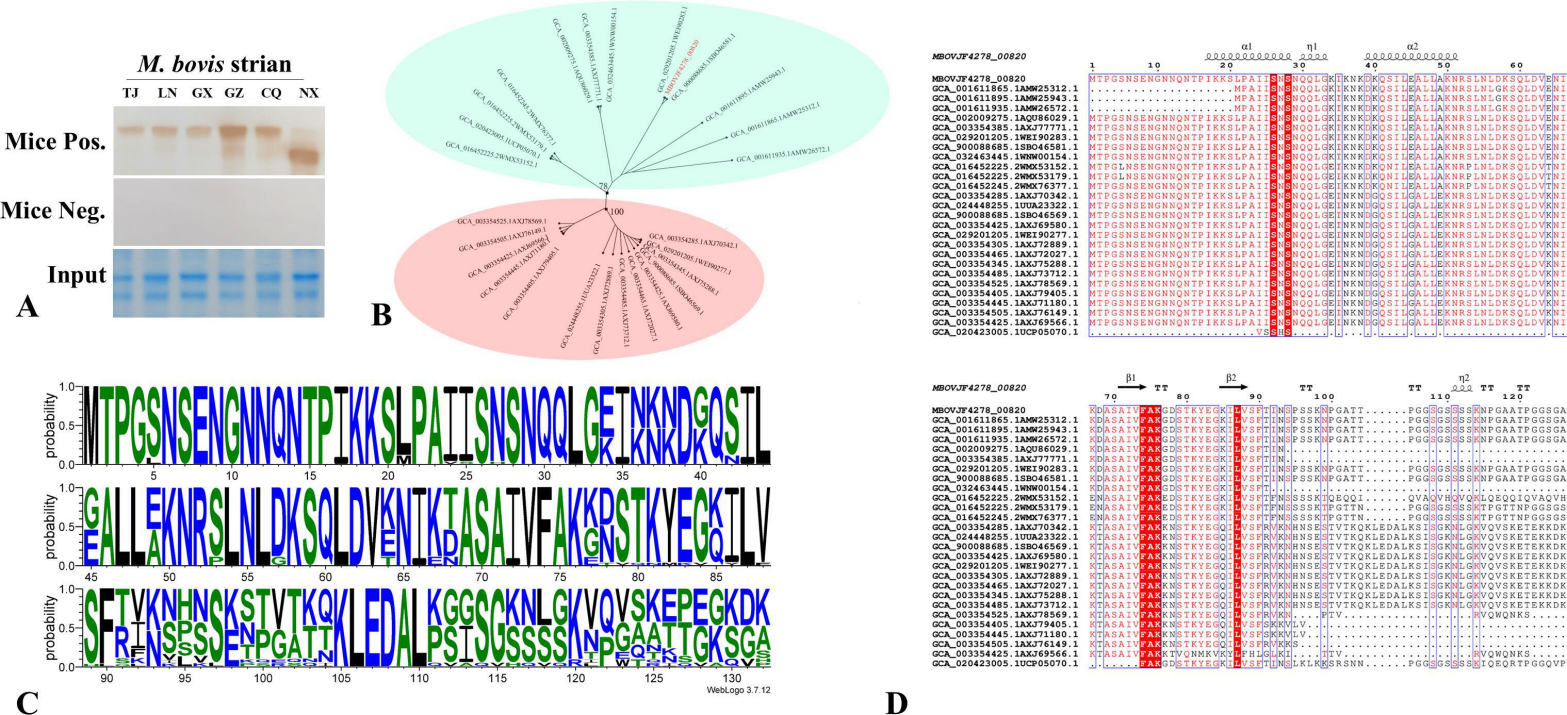
Conservation analysis of the *MBOVJF4278_00820*-encoded protein. (**A**) The anti-Pro820 polyclonal antibody was used to identify *M. bovis* strains from Tianjin (TJ), Liaoning (LN), Guangxi (GX), Guizhou (GZ), Chongqing (CQ), and Ningxia (NX), and reactive proteins were detected in all the aforementioned strains; (**B**) a refined phylogenetic tree depicting proteins from various *M. bovis* strains with homology exceeding 70% to the *MBOVJF4278_00820*-encoded protein; (**C**) the Weblogo analysis illustrates the conservation of the *MBOVJF4278_00820*-encoded protein, with analysis indicating that the first 90 amino acids exhibit a higher degree of conservation; (**D**) the ESPript was used to display the sequence alignment results of the *MBOVJF4278_00820*-encoded protein with the homologous proteins from different strains of *M. bovis*.

Western blotting was used to analyze the presence of the *MBOVJF4278_00820*-encoded protein in five strains of *M. bovis* isolated from different provinces in China. The immunoblotting results showed that a single band could be detected in five strains of bacteria using mouse anti-Pro820 serum, while no band could be detected with the pre-immune serum. We also found that the protein size detected in the *M. bovis* NX strain was smaller than that of other strains, indicating that there may be mutations in the *MBOVJF4278_00820* gene of this strain (Fig. 5A).

A comprehensive BLAST search was performed for the *MBOVJF4278_00820*-encoded protein across various strains of *M. bovis* within the NCBI database, successfully identifying matches in 47 distinct strains. Among these strains, 61 proteins exhibited a significant match with the *MBOVJF4278_00820*-encoded protein. Notably, 22 proteins demonstrated remarkable sequence homology exceeding 80%. In addition, the homology of four proteins is between 70% and 80%, while the homology of 35 proteins is between 70% and 30% (Table S1). Due to the significant difference between 35 proteins with homology below 70% and *MBOVJF4278_00820*-encoded protein, further analysis will not be conducted. To analyze the conservatism between the *MBOVJF4278_00820*-encoded protein and the remaining 26 proteins, sequence alignment was performed between these 26 matched proteins and the *MBOVJF4278_00820*-encoded protein using the MAFFT module (MAFFT< EMBL-EBI). The alignment results were used in IQ-TREE software for tree analysis, and it was found that these proteins could be divided into two main groups (Fig. 5B). Utilizing the WebLogo online server (WebLogo 3-About [threeplusone.com]), we analyzed the alignment of the *MBOVJF4278_00820*-encoded protein homologs across various strains. The results revealed a high degree of amino acid conservation at corresponding positions within these proteins, with the top 90 amino acids exhibiting particularly strong conservation (Fig. 5C). The ESPript’s analysis of the sequence also confirmed the conservation of the first 90 amino acid regions of these proteins (Fig. 5D).

## DISCUSSION

Heparin is a widely present sulfated glycosaminoglycan found in all types of tissues and cells. Heparin sulfate is synthesized in the Golgi matrix, and various modifications are carried out during the synthesis process, and the modification reaction does not occur uniformly on the chain (31). These modifications provide a material basis for the binding of heparin to different proteins. Heparin, as an important component of pathogen adhesion to host cells, has been confirmed in many pathogens, such as *Mycoplasma hyopneumoniae, Candida albicans*, and severe acute respiratory syndrome coronavirus 2 (32–34). Moreover, heparin may also be associated with the internalization and pathogenesis of pathogens (10). In this study, we initially discovered that the *MBOVJF4278_00820*-encoded protein can bind to host cells and further demonstrated that this protein can bind to host cell membrane components, ultimately confirming its binding to heparin. In addition to the adhesins identified in this article, MbfN and MilA, among the known adhesions of *M. bovis,* also have heparin-binding ability (12, 17). This indicates that heparin also plays a crucial role in the adhesion and colonization of *M. bovis*. Considering its wide distribution, heparin may have special significance in invading different tissues and cells during infection with *M. bovis*.

Infection with *M. bovis* can lead to different clinical signs, such as pneumonia, arthritis, and mastitis (1, 9, 35). Multiple clinical features are often associated with the invasiveness of various tissue cells. This study confirms that the *MBOVJF4278_00820*-encoded protein can bind not only to EBL cells but also to MDBK cells, indicating that this protein may play a role in the colonization of different cells. For the *M. bovis* adhesins, the function of adhesins was usually verified using EBL cells, except for the MbfN and the *MBOV_0503* encoding protein, which employed MDBK cells (12, 25). Considering that *M. bovis* causes different diseases in diverse tissues and organs of the host, the adhesions of this pathogen to distinct host cells may be different. This phenomenon was observed when identifying the cell adhesion ability of the VPS protein. When using monoclonal antibodies targeting the VSP protein to block the adhesion of *M. bovis*, certain antibodies can block the adhesion of *M. bovis* to EBL cells but do not affect the adhesion of *M. bovis* to BBE cells (23). In the present study, the Pro820 can bind to both EBL and MDBK cells, indicating that this adhesin may play a role in multiple cell species. The adhesion of *M. bovis* to host cells is achieved through various proteins, and this adhesion has functional redundancy. The superfluity of this function provides a basis for the immune escape of *M. bovis* and has special significance in the persistent infection of *M. bovis*. Moreover, the infection of *M. bovis* against different host cells demonstrates the complexity of its adhesion proteins and mechanisms. There are still many unknown fields for the adhesion of *M. bovis*. Such as the novel functions except adhesion, the binding component of host cells, and the roles in the pathogenesis process.

After being infected with *M. bovis*, in many cases, there are no obvious clinical symptoms, so its pathogenic role is often overlooked in clinical diagnosis. However, in recent years, more attention has been paid to this infamous animal pathogenic microorganism. In addition to being pathogenic alone, *M. bovis* is also one of the crucial pathogens of bovine respiratory syndrome and mastitis (8, 36). A vaccine is an effective tool for controlling infectious diseases. Unfortunately, although a large amount of research has been conducted on the vaccine against *M. bovis*, the progress of it is not satisfactory. People have tried inactivated vaccines, attenuated vaccines, subunit vaccines, and even cell membrane component vaccines, and only a few of these vaccines can reduce the degree of lung lesions to some extent. Some efforts not only fail to have a protective effect but also worsen clinical signs in the bacteria challenge experiment (37). Considering that the adhesion of pathogens is the first step of infection, using identified multiple adhesins as immune components and combining reverse vaccinology to develop the *M. bovis* vaccine may still be one of the research options worth trying. The rapid development of bioinformatics, especially the continuous progress of reverse vaccinology, is likely to provide new opportunities for the study of *M. bovis* vaccines. Many specific antibodies against *M. bovis* adhesins exhibit inhibitory ability against pathogen-host interactions. The adhesin-specific antibodies identified in this study also presented partial inhibition of *M. bovis* adhesion to host cells. Moreover, naturally infected bovine serum can significantly block the binding of the Pro820 protein to heparin. This indicates that the *MBOVJF4278_00820*-encoded protein is a potential subunit vaccine component.

This study employed various methods to identify an adhesin of *M. bovis*, but there are still some shortcomings. Although we confirmed that antibodies against the *MBOVJF4278_00820*-encoded protein can reduce the adhesion of *M. bovis* to host cells, a more direct method to prove the role of this protein in infection is gene knockout. Unfortunately, we lack the technology for specific gene mutation of *M. bovis*, and thus are unable to analyze the adhesion ability after knockout of the *MBOVJF4278_00820* gene in the strain used in this study. Another point to note is the identification of the natural infection serum reaction region of the *MBOVJF4278_00820*-encoded protein. In this study, we used western blotting to analyze the antigenicity of the truncated expressed region. We found that the region reacting with the positive serum is not in the same position as the heparin-binding region, although from the tertiary structure of the protein, the two segments are indeed adjacent, and antibodies may block the binding of heparin to the *MBOVJF4278_00820*-encoded protein through steric hindrance. However, there is also a possibility that conformational epitopes may play an important role. Due to the complex composition of the positive serum itself and the complexity of identifying conformational epitopes, we did not identify the conformational epitopes of the *MBOVJF4278_00820*-encoded protein.

*M. bovis* adhesins play a vital role in the colonization and invasion of host cells. Although multiple adhesins have been identified, the depth and breadth of these researches are far from sufficient. Therefore, it is crucial to intensify research on *M. bovis* adhesion and invasion mechanisms. This will facilitate a better comprehension of its pathogenic mechanism and serve as a reference for enhancing prevention and control methods.

## MATERIALS AND METHODS

### Bacterial strains, cells, and culture conditions

*M. bovis* strains of TJ, LN, GX, CQ, and NX were bacteria isolated from Tianjin, Liaoning, Guangxi, Chongqing, and Ningxia of China. These bacteria, which were preserved in our laboratory, were cultivated to the mid-logarithmic growth phase in *M. bovis* broth (pleuropneumonia-like organism [PPLO] medium containing 20% equine serum, 10% yeast extract, 0.125 mg/mL thallium acetate, and 200 IU/mL penicillin) (38). EBL cells and MDBK cells were cultured in Dulbecco’s modified Eagle medium with 10% heat-inactivated fetal bovine serum (Gibco), 100 µg/mL streptomycin (Gibco), and 100 U/mL penicillin (Gibco). These cells were incubated at 37°C in 5% CO_2_.

### Plasmid construction and protein expression

Genomic DNAs of *M. bovis* TJ strains were extracted using the Biospin Cell Genomic DNA Extraction Kit (BioFLUX). The DNA sequence of *MBOVJF4278_00820* from *M. bovis* strain JF4278 genome (GenBank: LT578453.1) was used to design primer pairs incorporating a homologous arm of the pET-28a vector. The Primers were named 00,820F and 00,820R (Table 1). Then, the *MBOVJF4278_00820* gene was cloned into the pET-28a vector with the ClonExpress II One Step Cloning Kit (Vazyme) according to the manufacturer’s instructions. After inducing the expression of this recombinant plasmid, the obtained protein is named Pro820. The whole and truncated expression genes encoding the amino acid regions 1-126, 1-40, 31-70, 61-100, and 91-126 of the *MBOVJF4278_00820*-encoded protein were synthesized and cloned into pGEX-4T-1 vector by Comate Biotech Company (Jilin, China) and were named as Pro820_GST_, Pro820_GST1-40_, Pro820_GST31-70_, Pro820_GST61-100_, and Pro820_GST91-126_, respectively. The gene encoding the initial 70 amino acids of the Pro820 protein was successfully cloned into the prokaryotic expression vector pGEX-4T-1 using the P1-70F/R primer pair. This construct was designated as pGEX-T-70 (Table 1). The protein induced by the expression of recombinant plasmid pGEX-T-70 is named Pro820_1-70GST_. Take pGEX-T-70 as a template, the amino acids at positions 34, 36, 38, and 40 were individually or collectively mutated to Alanine (Ala) using the primers Mu34F/R, Mu36F/R, Mu38F/R, and Mu40F/R (Table 1). The resulting mutants were named Mu34, Mu36, Mu38, Mu40, and MuAll, respectively.

**TABLE 1:** Primers for recombinant protein^*a*^

Primer names	Sequence (5′−3′)
00,820F	*cagcaaatgggtcgcggatcc*ATGACACCAGGCTCAAATTCTGA
00,820R	*acggagctcgaattcggatcc*GGCACCTGAGCCACCTGG
Mu34F	CCAACAATTAGGTgcAATTAAGAACAAAGACAAACAAAGCAT
Mu34R	ATTgcACCTAATTGTTGGTTACTATTGCTAATAATA
Mu36F	TTgcGAACAAAGACAAACAAAGCATCTTGGAAG
Mu36R	GTTTGTCTTTGTTCgcAATTTTACCTAATTGTTGGTTACTATTGC
Mu38F	GAACgcAGACAAACAAAGCATCTTGGAAGCATT
Mu38R	CTTTGTTTGTCTgcGTTCTTAATTTTACCTAATTGTTGGTTACTAT
Mu40F	GACgcACAAAGCATCTTGGAAGCATTATTAGCA
Mu40R	CAAGATGCTTTGTgcGTCTTTGTTCTTAATTTTACCTAATTGTTG
MuAllF	cAATTgcGAACgcAGACgcACAAAGCATCTTGGAAGCATTATTA
MuAllR	cGTCTgcGTTCgcAATTgcACCTAATTGTTGGTTACTATTGCTAATAATA
P1-70F	*gatctggttccgcgtggatcc*ATGACACCAGGCTCAAATTCTGA
P1-70R	*acccgggaattccggggatcc*TGATGCATCTTTTATATTTTCAACATCT

^*a*^ Lowercase letters indicate mutated nucleotides. Italic lowercase letters represent homologous arms.

These recombinant plasmids were routinely transformed into *E. coli* BL21(DE3) cells. The expression of the recombinant fusion proteins was induced in a logarithmic-phase culture of the transformants by the addition of isopropyl-β-D-1-thiogalactopyranoside (Sigma) to a final concentration of 0.5 mM at 16°C for 24 hours. Recombinant proteins fused with HIS or GST tags were purified using the Ni-NTA HIS-bind resin affinity chromatography purification column (Cytiva) or the glutathione resin column (ThermoFisher), respectively, following the manufacturer’s recommended methods. The purified protein was analyzed using SDS-PAGE and western blotting. Purified protein concentration was quantified using a BCA protein assay kit (Solarbio) according to the manufacturer’s instructions. The Hyp72 (GST) protein is an *Mmm* bacterial protein expressed with a GST tag, which is preserved in our laboratory.

### Polyclonal antibody production

Antiserum to Pro820 was raised in BALB/c mice. Briefly, the mice were subcutaneously immunized with 50 µg of purified antigen emulsified in Freund’s complete adjuvant (Sigma-Aldrich). After 14 days, the mice were boosted with 50 µg of antigen mixed with Freund’s incomplete adjuvant (Sigma-Aldrich). Afterward, blood was collected from the tail tip of mice every 14 days to determine the serum antibody titer, and booster immunization should be conducted until the serum titer reaches the expected level. Then, a large amount of blood was collected to prepare the serum and stored at −20°C. To verify the specificity of the prepared serum, western blotting was performed to assess the reactivity of the mouse anti-Pro820 serum with Pro820 protein, GST tag, unrelated proteins fused with GST tag, and membrane components of MDBK cell and EBL cell membranes, respectively.

### Whole-cell lysates, membrane, and cytoplasmic protein preparation

*M. bovis* in the mid-logarithmic phase was harvested by centrifugation at 12,000 × *g* for 5 minutes. The cell pellets were washed with PBS three times. Membrane and cytoplasmic proteins of *M. bovis* were extracted by the ProteoExtract Transmembrane Protein Extraction Kit (Sigma). The whole protein of the *M. bovis* TJ strain was obtained by ultrasonic lysis. The membrane proteins of EBL and MDBK cells were extracted using membrane protein extraction kits (Solarbio). The concentration of all proteins was determined by the BCA protein quantification kit (Solarbio).

### Western blotting assay

The western blotting was performed as described previously with a slight modification (16). Some results of western blotting were analyzed for grayscale using Image J software (ImageJ).

For the *MBOVJF4278_00820*-encoded protein antigenicity analysis, the purified Pro820 was electrophoretically separated on an SDS-PAGE and then transferred onto the nitrocellulose (NC) film (GE). The film was blocked with 5% gelatin in PBS for 2 hours. The blocked blot was incubated with bovine sera (diluted 1:100 in PBS) for 2 hours. Later, the bound antibodies were detected by incubation for 1 hour with horseradish peroxidase (HRP)-labeled rabbit anti-bovine IgG (diluted 1:8,000) (Sigma). Color development was performed using the DAB Substrate kit (Solarbio).

For subcellular localization or conservation analysis, 2 µg of total, membrane, and cytoplasmic proteins were separated by 12% SDS-PAGE and transferred onto NC film. The unoccupied sites were blocked with 5% gelatin in PBS for 2 hours, followed by incubation with mouse anti-Pro820 polyclonal antibodies (diluted 1:1,000 in PBS) for 2 hours. The 5,000-fold dilution of HRP-labeled goat anti-mouse IgG (H+L) antibodies was used as secondary antibodies (ZSGB-BIO). Color development was performed using the DAB Substrate kit.

For heparin binding examination, proteins were separated by 12% SDS-PAGE and transferred onto NC film. The film was blocked with 5% gelatin in PBS for 2 hours. Followed by and incubated with heparin-biotin sodium salt (Sigma). Then, 50 µg/mL of heparin-biotin (Sigma) was incubated with the film, followed by the PEROXIDASE-CONJUGATE GOAT ANTI-BIOTIN antibody (Sigma). The blot was developed with a DAB substrate kit according to the manufacturer’s instructions. The serum-blocking heparin-binding assay is similar to the heparin adhesion assay, except that the membrane was incubated with bovine-derived *M. bovis* positive serum or mouse anti-Pro820 protein serum at room temperature for 2 hours before the incubation with heparin.

### ELISA

To verify the adhesion of recombinant proteins to cells, 10 µg/mL of membrane component in carbonate buffer (18 mM NaHCO_3_, 27 mM Na_2_CO_3_ pH 9.6) was coated onto an ELISA plate (CORNING) at 4°C for 12 hours. Unoccupied sites were blocked with 5% gelatin at 37°C for 2 hours. Different concentrations of purified Pro820 (10, 5, 2.5, 1.25, 0.625, 0.3125, 0.15625 µg/mL) were added and incubated at 37°C for 1 hour. The mouse anti-Pro820 polyclonal antibodies (1:5,000) were employed to react with the bound protein. Finally, HRP-labeled goat anti-mouse IgG (H+L) (ZSGB-BIO) (1:4,000) was added and incubated at 37°C for 1 hour. Then, the TMB (Sigma) staining was used to detect the proteins adhering to the cell membrane components. Three independent experiments were performed.

For positive serum block analysis, the ELISA plate was coated with the EBL/MDBK cell membrane proteins and blocked with gelatin as mentioned above. Twenty-five times dilution of *M. bovis* positive serum was mixed with 25 µg/mL of the Pro820 and placed at 37°C for 1 hour. The mixture was transferred to the ELISA plate and incubated at 37°C for 1 hour. After washing three times with PBST, the mouse anti-HIS antibodies (1:2,000) were added and incubated at 37°C for 1 hour. Followed HRP-labeled goat anti-mouse IgG incubation. Then, the OD_450 nm_ of absorbance value was measured after TMB was added. In this experiment, the negative bovine serum was set as a control. Three independent experiments were performed. An unpaired *t*-test analysis was performed for different serum treatment groups.

To verify the ability of the Pro820 binding heparin. Ninety-six well plates were coated with purified Pro820 (5 µg/mL) in carbonate buffer at 4°C for 12 hours and then blocked with gelatin as the previously described method. After being washed three times, the wells were incubated with varying concentrations of heparin-biotin sodium salt (10^2^, 10^1^, 10^0^, 10^−1^, 10^−2^, 10^−3^, 10^−4 ^μg/mL) at 37°C for 1 hour. Unbonded heparin was removed by washing three times with PBST, and PEROXIDASE-CONJUGATE GOAT ANTI-BIOTIN antibody was added and incubated at 37°C for 1 hour to detect binding heparin. Fifty microliters of TMB was added to each well, and ELISA plates were kept at 37°C for 10 minutes. After that, the reaction was terminated with 2 M H_2_SO_4_, and the values of OD_450 nm_ absorbance were measured.

### Dot-ELISA

The proteins were fixed onto the NC membrane without denaturation. The NC film was sealed with 5% gelatin at room temperature for 2 hours. After that, the film was incubated with biotinylated heparin (50 µg/mL) at room temperature for 2 hours, and the PEROXIDASE-CONJUGATE GOAT ANTI-BIOTIN antibodies (1:2,000) were employed for the detection of the binding heparin at temperature for 1 hour. Finally, the color was developed with a DAB substrate kit. An input control with Ponceau S staining was also set up to confirm that the protein was successfully adsorbed onto the NC membrane.

### Indirect immunofluorescence assay

To confirm that the *MBOVJF4278_00820*-encoded protein is located on the surface of *M. bovis*, the subcellular localization of the protein was determined using IFA (15). In short, *M. bovis* that grew to the logarithmic growth stage was harvested and washed three times with PBS. The intact *M. bovis* cells (10^10^ CCU) were incubated with mouse anti-Pro820 serum and pre-immune serum. Samples were incubated with fluorescein isothiocyanate (FITC)-labeled goat anti-mouse IgG (whole molecule) (ZSGB-BIO) at 37°C for 1 hour after washing three times with PBST. The cells were visualized under a fluorescence microscope (AMG) after another three washes.

To validate the direct adhesin of the *MBOVJF4278_00820*-encoded protein to host cells, the EBL/MDBK cells were cultured for 24 hours and washed three times with PBS, then fixed with 4% paraformaldehyde at 4°C for 30 minutes. The cells were blocked with 1% bovine serum albumin (BSA) at 37°C for 2 hours. Subsequently, the cells were incubated in 1 mL of PBS containing 100 µg of Pro820. The cells incubated with PBS without the Pro820 or the pre-immune serum were set as the control. To ensure the activity of the recombinant protein, the Pro820 adhesion validation was performed immediately after purification. The bound protein was stained with mouse anti-Pro820 polyclonal antibodies at 37°C for 1 hour, and FITC-labeled goat anti-mouse IgG antibody, while cell nuclei were stained with 4’,6-diamidino-2-phenylindole. Immunofluorescence was examined by a confocal microscope.

In the experiment where the serum of naturally infected *M. bovis* blocks the adhesion of Pro820 protein to host cells, the MDBK cells were fixed and blocked as mentioned above. Two hundred micrograms per microliter of the Pro820 protein were mixed with an equal volume of fivefold diluted naturally infected bovine serum, and the mixture was placed at 37°C for 1 hour. Next, this mixture was incubated with MDBK cells at 37°C for 1 hour, the 2,000-fold dilution of mouse anti-Pro820 polyclonal antibodies was incubated with the bound protein, and 100-fold dilution of FITC-labeled goat anti-mouse IgG antibodies was employed for immunofluorescence examination. This experiment simultaneously included a FITC antibody control group, a PBS-blocking group, and a negative serum-blocking group. Immunofluorescence was examined by a confocal microscope.

In the heparin blockade experiment, the confluent monolayers of EBL and MDBK cells are cultured. Cells are fixed with 4% formaldehyde and blocked with 1% BSA. The Pro820 protein (200 µg/mL) was mixed with heparin (1 mg/mL) in equal volumes and incubated at 37°C for 1 hour. Concurrently, the Pro820 protein (200 µg/mL) was mixed with PBS in equal volumes as a control. The heparin-blocked samples and control samples are then reacted with fixed EBL and MDBK cells at 37°C for 1 hour. Subsequently, the same procedure as in the experiment for endogenous heparin elimination is followed by adding the mouse anti-Pro820 serum and FITC-labeled goat anti-mouse antibody to the cells. Finally, the fluorescence of cells in different treatment groups is observed and recorded.

In the experiment of endogenous heparin elimination, the MDBK cells were taken as a model and treated with Heparinase I (Sigma) and Heparinase III (Sigma) to remove endogenous heparin. The digestion of heparinase follows the manufacturer’s instructions. Briefly, four groups of MDBK cells were fixed using the aforementioned method. The first group was subjected to treatment with heparinase I, the second group with heparinase III, the third group received a combined treatment of both enzymes, and the fourth group served as a control with the addition of PBS. These cells were incubated with different reagents at 31°C for 24 hours. The cells were blocked with BSA by the previously described method, followed by incubation with 100 µL (50 µg/mL) of the Pro820 protein at 4°C for 1 hour, and then 2,000-fold diluted mouse anti-Pro820 protein immune serum was added and incubated at room temperature for 1 hour. Next, a 100-fold diluted FITC-labeled goat anti-mouse antibody (ZSGB-BIO) was added and incubated at room temperature for 1 hour. Finally, the results were observed and recorded under a fluorescence microscope. Before each of the aforementioned procedures, a five-time wash with PBST was conducted.

### Adherence inhibition assay of *M. bovis* TJ strains

The inhibition of *M. bovis* adhesion to MDBK cells by the mouse anti-Pro820 polyclonal antibody was performed according to previously reported methods with slight modifications (15). Briefly, MDBK cells were seeded into a 12-well culture plate (2 × 10^5^ cells/well) and grown to a confluent monolayer. Subsequently, the cells were infected with *M. bovis*, which had been pre-treated with mouse pre-immune serum or anti-Pro820 serum at 37°C for 1 hour. Then, the samples were washed five times with PBST to remove unbound *M. bovis*. These cells were harvested after being digested with 0.25% trypsin in MEM. The CFU of bounded *M. bovis* was detected by plating on modified PPLO agar dishes for bacterial colony counting. Three independent experiments were performed. An unpaired *t*-test was performed for the CFU of different groups with GraphPad Prism software.

### Tertiary structure prediction and conservation analysis

The I-TASSER online server (I-TASSER server for protein structure and function prediction [zhanggroup.org]) was employed for tertiary structural prediction of the *MBOVJF4278_00820*-encoded protein.

The conservation of *MBOVJF4278_00820*-encoded protein was analyzed using the NCBI database. Firstly, the BLAST module was used to compare and analyze the encoded proteins of different strains of *M. bovis*, and proteins with homology to the *MBOVJF4278_00820*-encoded protein were preliminary screened. Next, the proteins with an Identity% more than 70% to the *MBOVJF4278_00820*-encoded protein were selected. Perform sequence alignment between the *MBOVJF4278_00820*-encoded protein and the screened proteins using the MAFFT online server (MAFFT<EMBL-EBI) (39). The length processing based on the *MBOVJF4278_00820*-encoded protein was performed for these alignment sequences by the BioEdit software, and then a phylogenetic tree was constructed by the IQ-TREE ML tools (40, 41). Additionally, the sequence alignment results are displayed using the ESPript 3.0 online server (ESPript 3 [ibcp.fr]) (42). Furthermore, for homologous proteins of the *MBOVJF4278_00820*-encoded protein in different strains, the conservation of amino acids at different positions was analyzed using the WebLogo program (WebLogo - Create Sequence Logos [berkeley.edu]) (43).

### Bio-layer interferometry analysis

The affinity of Pro820_1-70 GST_, Mu34, Mu36, Mu38, and Mu40 for heparin was determined using the Octet RED96e System (ForteBio). The detailed procedural steps are outlined in Table 2. Briefly, all samples are diluted in black 96-well microplates (Microplate 96 Well, Greiner). Throughout each step, the working volume per well is maintained at 200 µL, with all reactions conducted at a constant temperature of 25°C. Prior to each assay, the GST biosensor tip (fortebio) is pre-equilibrated in 200 µL of PBS for a minimum of 10 minutes, followed by an equilibrium period of 120 seconds using PBS buffer. Protein binding to the biosensor is then initiated for a duration of 300 seconds, after which the system is equilibrated again in PBST for 120 seconds. The biosensor is subsequently exposed to heparin of varying concentrations (400, 800, 1,600, 3,200, 6,400 µg/mL) for a binding reaction period of 300 seconds. Finally, the biosensor is subjected to a dissociation phase in PBST for 300 seconds. Upon completion of the experiment, the raw data is imported into Excel for further analysis using Octe Software (Data Analysis 9.0).

**TABLE 2:** BLI measuring protocol for heparin-protein interactions

Step	Step name	Sample type	Position	Step time (s)
1	Baseline	PBS	Tube	120
2	Loading	Protein	Drop	300
3	Baseline2	PBST	Tube	120
4	Association	Heparin	Drop	300
5	Dissociation	PBSTs	Tube	300

### Statistical analysis

The R programming language (v4.4.3) was utilized for conducting all statistical analyses. Initially, the Shapiro-Wilk test was performed to assess data normality, followed by Bartlett’s test to verify homogeneity of variances. After confirming that the data set satisfied both normality and homoscedasticity assumptions, we conducted a one-way analysis of variance to examine group differences. To ensure rigorous multiple comparisons, post hoc analyses were subsequently carried out using Tukey’s honest significant difference test. All graphs were plotted as boxplots with overlaid data points with ggplot2 (v3.5.1). In the context of all analyses, a *P*-value of less than 0.05 was deemed to indicate statistical significance (*), a *P*-value less than 0.01 was considered to represent a highly significant difference (**), and a *P*-value less than 0.001 was regarded as denoting an extremely significant difference (***).

## Data Availability

All data supporting the findings of this study are available from the corresponding author upon reasonable request.

## SUPPLEMENTAL MATERIAL

Table S1 iai.00606-24-s0001.docx BLAST analysis of MBOVJF4278-00820-encoded protein under *M. bovis* species in the NCBI database.

ASM does not own the copyrights to Supplemental Material that may be linked to, or accessed through, an article. The authors have granted ASM a non-exclusive, world-wide license to publish the Supplemental Material files. Please contact the corresponding author directly for reuse.
