# Supplementary material for: MBOVJF4278_00820 encodes a novel cytoadhesin of Mycoplasma bovis binding to heparin
Source: Infect Immun. 2025 Apr 23;93(5):e00606-24. doi: 10.1128/iai.00606-24 (PMC12070734; doi:10.1128/iai.00606-24)
Supplement: Table S1 — BLAST analysis of MBOVJF4278-00820-encoded protein under M. bovis species in the NCBI database. [file iai.00606-24-s0001.docx]

Supplementary Table S1 BLAST analysis of *MBOVJF4278-00820*-encoded protein under *M. bovis* species in the NCBI database

| **query acc.ver** | **subject acc.ver** | **% identity** | **alignment length** | **mismatches** | **gap opens** | **q. start** | **q. end** | **s. start** | **s. end** | **e-value** | **bit score** |
| --- | --- | --- | --- | --- | --- | --- | --- | --- | --- | --- | --- |
| GCA_900088685.1 | SBO46581.1 | 100 | 126 | 0 | 0 | 1 | 126 | 1 | 126 | 7.79E-85 | 243 |
| GCA_029201205.1 | WEI90283.1 | 100 | 126 | 0 | 0 | 1 | 126 | 173 | 298 | 6.05E-83 | 244 |
| GCA_001611935.1 | AMW26572.1 | 98.113 | 106 | 2 | 0 | 21 | 126 | 1 | 106 | 1.42E-68 | 201 |
| GCA_001611895.1 | AMW25943.1 | 98.113 | 106 | 2 | 0 | 21 | 126 | 1 | 106 | 1.42E-68 | 201 |
| GCA_001611865.1 | AMW25312.1 | 98.113 | 106 | 2 | 0 | 21 | 126 | 1 | 106 | 1.42E-68 | 201 |
| GCA_016452245.2 | WMX76377.1 | 76.056 | 142 | 18 | 2 | 1 | 126 | 160 | 301 | 1.88E-61 | 190 |
| GCA_016452225.2 | WMX53179.1 | 75.352 | 142 | 19 | 2 | 1 | 126 | 217 | 358 | 4.36E-60 | 188 |
| GCA_003354385.1 | AXJ77771.1 | 100 | 93 | 0 | 0 | 1 | 93 | 173 | 265 | 2.62E-59 | 183 |
| GCA_002009275.1 | AQU86029.1 | 100 | 93 | 0 | 0 | 1 | 93 | 173 | 265 | 2.62E-59 | 183 |
| GCA_032463445.1 | WNW00154.1 | 100 | 91 | 0 | 0 | 1 | 91 | 217 | 307 | 3.87E-57 | 179 |
| GCA_016452225.2 | WMX53152.1 | 89.796 | 98 | 10 | 0 | 1 | 98 | 217 | 314 | 2.60E-54 | 174 |
| GCA_003354525.1 | AXJ78569.1 | 80.612 | 98 | 19 | 0 | 1 | 98 | 130 | 227 | 3.23E-48 | 154 |
| GCA_003354505.1 | AXJ76149.1 | 84.783 | 92 | 14 | 0 | 1 | 92 | 160 | 251 | 1.61E-47 | 153 |
| GCA_003354445.1 | AXJ71180.1 | 85.714 | 91 | 13 | 0 | 1 | 91 | 160 | 250 | 4.96E-47 | 152 |
| GCA_003354405.1 | AXJ79405.1 | 85.714 | 91 | 13 | 0 | 1 | 91 | 160 | 250 | 4.96E-47 | 152 |
| GCA_003354485.1 | AXJ73712.1 | 84.783 | 92 | 14 | 0 | 1 | 92 | 130 | 221 | 6.27E-47 | 152 |
| GCA_003354465.1 | AXJ72027.1 | 84.783 | 92 | 14 | 0 | 1 | 92 | 130 | 221 | 2.73E-46 | 151 |
| GCA_003354305.1 | AXJ72889.1 | 84.783 | 92 | 14 | 0 | 1 | 92 | 130 | 221 | 2.73E-46 | 151 |
| GCA_003354345.1 | AXJ75288.1 | 84.783 | 92 | 14 | 0 | 1 | 92 | 130 | 221 | 3.73E-46 | 151 |
| GCA_029201205.1 | WEI90277.1 | 80.612 | 98 | 19 | 0 | 1 | 98 | 100 | 197 | 3.22E-45 | 151 |
| GCA_900088685.1 | SBO46569.1 | 80.612 | 98 | 19 | 0 | 1 | 98 | 100 | 197 | 5.33E-45 | 151 |
| GCA_024448255.1 | UUA23322.1 | 80.612 | 98 | 19 | 0 | 1 | 98 | 100 | 197 | 5.33E-45 | 151 |
| GCA_003354285.1 | AXJ70342.1 | 80.612 | 98 | 19 | 0 | 1 | 98 | 100 | 197 | 5.33E-45 | 151 |
| GCA_003354425.1 | AXJ69580.1 | 80.612 | 98 | 19 | 0 | 1 | 98 | 110 | 207 | 1.93E-44 | 151 |
| GCA_003354425.1 | AXJ69566.1 | 75 | 92 | 23 | 0 | 1 | 92 | 96 | 187 | 2.27E-38 | 128 |
| GCA_020423005.1 | UCP05070.1 | 72 | 50 | 14 | 0 | 74 | 123 | 7 | 56 | 6.40E-10 | 51.6 |
| GCA_900088685.1 | SBO45811.1 | 38.947 | 95 | 48 | 5 | 32 | 123 | 53 | 140 | 5.09E-08 | 50.1 |
| GCA_032463445.1 | WNW00240.1 | 38.947 | 95 | 48 | 5 | 32 | 123 | 53 | 140 | 5.09E-08 | 50.1 |
| GCA_029201205.1 | WEI90379.1 | 38.947 | 95 | 48 | 5 | 32 | 123 | 53 | 140 | 5.09E-08 | 50.1 |
| GCA_024448255.1 | UUA23417.1 | 38.947 | 95 | 48 | 5 | 32 | 123 | 53 | 140 | 5.09E-08 | 50.1 |
| GCA_021497165.1 | UJB27265.1 | 38.947 | 95 | 48 | 5 | 32 | 123 | 53 | 140 | 5.09E-08 | 50.1 |
| GCA_021497145.1 | UJB26498.1 | 38.947 | 95 | 48 | 5 | 32 | 123 | 53 | 140 | 5.09E-08 | 50.1 |
| GCA_021497125.1 | UJB25704.1 | 38.947 | 95 | 48 | 5 | 32 | 123 | 53 | 140 | 5.09E-08 | 50.1 |
| GCA_021497085.1 | UJB24236.1 | 38.947 | 95 | 48 | 5 | 32 | 123 | 53 | 140 | 5.09E-08 | 50.1 |
| GCA_020423005.1 | UCP05159.1 | 38.947 | 95 | 48 | 5 | 32 | 123 | 53 | 140 | 5.09E-08 | 50.1 |
| GCA_020422965.1 | UCP03493.1 | 38.947 | 95 | 48 | 5 | 32 | 123 | 53 | 140 | 5.09E-08 | 50.1 |
| GCA_020422945.1 | UCP02645.1 | 38.947 | 95 | 48 | 5 | 32 | 123 | 53 | 140 | 5.09E-08 | 50.1 |
| GCA_016807745.1 | QRF87521.1 | 38.947 | 95 | 48 | 5 | 32 | 123 | 53 | 140 | 5.09E-08 | 50.1 |
| GCA_016807725.1 | QRF86697.1 | 38.947 | 95 | 48 | 5 | 32 | 123 | 53 | 140 | 5.09E-08 | 50.1 |
| GCA_016452245.2 | WMX75847.1 | 38.947 | 95 | 48 | 5 | 32 | 123 | 53 | 140 | 5.09E-08 | 50.1 |
| GCA_016452225.2 | WMX52591.1 | 38.947 | 95 | 48 | 5 | 32 | 123 | 53 | 140 | 5.09E-08 | 50.1 |
| GCA_011996705.1 | QIT09167.1 | 38.947 | 95 | 48 | 5 | 32 | 123 | 53 | 140 | 5.09E-08 | 50.1 |
| GCA_004792535.1 | QCA29752.1 | 38.947 | 95 | 48 | 5 | 32 | 123 | 53 | 140 | 5.09E-08 | 50.1 |
| GCA_003354525.1 | AXJ77900.1 | 38.947 | 95 | 48 | 5 | 32 | 123 | 53 | 140 | 5.09E-08 | 50.1 |
| GCA_003354505.1 | AXJ75422.1 | 38.947 | 95 | 48 | 5 | 32 | 123 | 53 | 140 | 5.09E-08 | 50.1 |
| GCA_003354485.1 | AXJ73029.1 | 38.947 | 95 | 48 | 5 | 32 | 123 | 53 | 140 | 5.09E-08 | 50.1 |
| GCA_003354465.1 | AXJ71315.1 | 38.947 | 95 | 48 | 5 | 32 | 123 | 53 | 140 | 5.09E-08 | 50.1 |
| GCA_003354445.1 | AXJ70477.1 | 38.947 | 95 | 48 | 5 | 32 | 123 | 53 | 140 | 5.09E-08 | 50.1 |
| GCA_003354425.1 | AXJ68924.1 | 38.947 | 95 | 48 | 5 | 32 | 123 | 53 | 140 | 5.09E-08 | 50.1 |
| GCA_003354405.1 | AXJ78705.1 | 38.947 | 95 | 48 | 5 | 32 | 123 | 53 | 140 | 5.09E-08 | 50.1 |
| GCA_003354385.1 | AXJ77102.1 | 38.947 | 95 | 48 | 5 | 32 | 123 | 53 | 140 | 5.09E-08 | 50.1 |
| GCA_003354365.1 | AXJ76283.1 | 38.947 | 95 | 48 | 5 | 32 | 123 | 53 | 140 | 5.09E-08 | 50.1 |
| GCA_003354345.1 | AXJ74602.1 | 38.947 | 95 | 48 | 5 | 32 | 123 | 53 | 140 | 5.09E-08 | 50.1 |
| GCA_003354305.1 | AXJ72169.1 | 38.947 | 95 | 48 | 5 | 32 | 123 | 53 | 140 | 5.09E-08 | 50.1 |
| GCA_002749575.1 | ATQ40000.1 | 38.947 | 95 | 48 | 5 | 32 | 123 | 53 | 140 | 5.09E-08 | 50.1 |
| GCA_002009275.1 | AQU85365.1 | 38.947 | 95 | 48 | 5 | 32 | 123 | 53 | 140 | 5.09E-08 | 50.1 |
| GCA_001043135.1 | AKO50278.1 | 38.947 | 95 | 48 | 5 | 32 | 123 | 53 | 140 | 5.09E-08 | 50.1 |
| GCA_001611895.1 | AMW25392.1 | 38.947 | 95 | 48 | 5 | 32 | 123 | 87 | 174 | 5.36E-08 | 49.7 |
| GCA_001611935.1 | AMW26023.1 | 38.947 | 95 | 48 | 5 | 32 | 123 | 87 | 174 | 5.52E-08 | 49.7 |
| GCA_001611865.1 | AMW24761.1 | 38.947 | 95 | 48 | 5 | 32 | 123 | 87 | 174 | 5.52E-08 | 49.7 |
| GCA_021497185.1 | UJB28016.1 | 37.895 | 95 | 49 | 5 | 32 | 123 | 53 | 140 | 1.86E-07 | 48.1 |
